# Pulmonary manifestations in Behçet disease: impaired natural killer cells activity

**DOI:** 10.1186/2049-6958-8-29

**Published:** 2013-04-04

**Authors:** Kamel Hamzaoui, Anissa Berraies, Wajih Kaabachi, Jamel Ammar, Agnès Hamzaoui

**Affiliations:** 1 Department of Basic Sciences, Division of Histology and Immunology, Medicine School of Tunis, Tunis El Manar University, 15 Rue DjebelLakdar, Tunis, 1007, Tunisia; 2 Department of respiratory diseases and the Unit Research “Homeostasis and Cell dysfunction (99/08-40), Division of Pulmonology, Tunis El Manar University and A. Mami Hospital, Ariana, Tunisia

**Keywords:** Behçet disease, Bronchoalveolar lavage, Granzyme, Inflammation, Natural killer cells, Perforin

## Abstract

**Background:**

Behçet’s disease (BD) is a systemic vasculitis with unknown aetiology, where, besides genetic predisposition, an immune dysregulation involving T and B lymphocytes and hyperactive neutrophils contribute to disease pathogenesis. The aim of this study was to determine the cytotoxicity of natural killer (NK) cells in bronchoalveolar lavage (BAL) from BD patients with pulmonary manifestations.

**Methods:**

BAL was performed in 27 patients with BD and pulmonary manifestations, 14 patients with Rheumatoid Arthritis (RA) and 23 healthy controls (HC). Related orphan receptor C (RORC) and forkheadbox P3 (FOXP3) mRNA transcript were determined in BAL by reverse transcription–polymerase chain reaction (RT-PCR). NK cells, NK cell cytotoxicity, and lymphokine-activated killer (LAK) activity against K562 cells were measured by flow cytometry. Proportions of NK precursors and expression of genes for IL-2 receptor β (IL-2Rβ; CD122), perforin, and granzyme in NK cells were measured by flow cytometry or RT-PCR.

**Results:**

The analysis of transcription factors revealed an increase in the RORC/FOXP3 ratio (Th17/Treg cells) in BAL from BD patients. Percentages of NK were significantly lower in BD than in RA patients and healthy controls. Purified NK cells derived from BD patients were found to have lower cytotoxicity and LAK activity than those from controls. This defect of NK cells in BD patients was related to down-regulation of perforin and granzyme expression in NK cells.

**Conclusion:**

In BD patients, the increased RORC/FOXP3 ratio indicated an inflammatory state of the lung. NK cells were decreased together with an impairment of their activity due to a defective expression of granzyme and perforin. These abnormalities possibly contribute to immune system dysregulation found in BAL of BD patients with pulmonary manifestations.

## Background

Behçet’s disease (BD) is a systemic vasculitis with unknown aetiology. Immune dysregulation involving T and B cells with hyperactive neutrophils, supposedly triggered by infectious agents, contribute to disease pathogenesis in addition to genetic predisposition [1]. Documentation of various atypical streptococcal species in oral flora of BD patients, clinical flares after dental procedures and a good response to antibacterial treatment have been considered as evidence for the role of *Streptococcus* in BD [2]. However, none of the microbial agents has been definitely proved to cause BD. Immunological disorders are important in BD pathogenesis [3]. T lymphocytes from patients with BD produced a particular pattern of inflammatory mediators when stimulated with a bacterial superantigen, and innate immunity was deeply investigated in BD patients [4].

In Behçet’s disease, vascular system involvement is the main cause of mortality. Pulmonary artery aneurysms, arterial and venous thrombosis, pulmonary infarction, recurrent pneumonia, bronchiolitis obliterans organized pneumonia, and pleurisy are the main features of pulmonary involvement in BD [5,6]. Inflammatory features characterize bronchoaveolar lavage (BAL) from BD patients with pulmonary involvement. B cell-activating factor of the TNF family (BAFF), an important regulator of B-cell survival and immunoglobulin class-switch recombination is increased in BD lung and contributes to immunoglobulin synthesis [7]. Both interleukin 18 (IL-18) and gamma interferon (IFN-γ), contribute to the local inflammatory response in BAL from BD patients [8]. Recently Toll-like receptors expressing cells and NOD-like receptors (NLRs) were found to synergize for the induction of proinflammatory cytokines in BAL from BD patients with pulmonary manifestations [9].

As major components of innate immunity, Natural killer (NK) cells not only exert cell-mediated cytotoxicity against tumour or infected cells, but also regulate other immune cells functions by secretion of cytokines and chemokines. Due to these effector functions, NK cells play a significant role in host defense against malignancies and certain viruses and they may also be important in the regulation of autoimmunity [10]. However, the effector function of NK cells must be exquisitely controlled in order to prevent inadvertent attack against self normal cells. Patients with active BD show impaired NK cytotoxicity [11-14]. Impaired NK cytotoxicity in first-degree relatives of BD patients was recently reported [14-16], which suggests that NK cell deficiency, may be a genetic determinant of BD.

The aim of the present study was to determine the expression of retinoid-related orphan receptor C (RORC) (Th17), forkheadbox P3 (FOXP3) (Treg) and the cytotoxicity of pulmonary NK cells in BD. We determined NK cell levels, NK cytotoxicity, and lymphokine-activated killer (LAK) activity in BAL of patients with BD. Proportions of NK precursors and expression of genes for IL-2 receptor β-chain (IL-2Rβ; CD122), perforin, and granzyme in NK cells were measured by flow cytometry or reverse transcription–polymerase chain reaction (RT-PCR).

## Methods

### Patients

The study group consisted of 27 BD patients (19 males, 8 females, age 34 ± 10 years; range 17–56 years) all fulfilling the international study group criteria for Behcet’s disease [17], with a disease duration ranging from 1 to 9 years (mean ± SD: 5.8 ± 3.4). Twenty three BD patients were never-smokers and 4 ex-smokers. All patients had active BD with pulmonary manifestations [8,9] including eye lesions (14 patients: 51.85%), oral ulcers (27 patients: 100%) , genital ulcers (18 patients: 66.67%), arthritis (16 patients: 59.25%), and vascular symptoms (12 patients: 44.45%). Pulmonary vascular abnormalities were as follows: asymptomatic functional abnormalities (8 patients), pulmonary artery aneurysm (6 patients), pulmonary artery embolism (9 patients), and pulmonary venous abnormalities (4 patients). They were treated with steroids and colchicine. Remission was defined when clinical manifestations were lost (eye lesions, oral and genital ulcers, and arthritis). Asymptomatic functional abnormalities diminished after corticosteroid treatment. Rheumatoid arthritis patients (RA: 10 men and 4 women; mean age: 46.2 ± 9.5 years; range: 42–50 years) acted as control disease. The control subjects consisted of 23 non-smokers (18 men and 5 women; mean age: 42.8 ± 7 years; range: 38–52 years) undergoing routine investigations for suspected bronchial carcinoma and whose chest X-ray, bronchial examination, and pulmonary function were normal. None of them had evidence of acute infection or chronic disease (e.g., other autoimmune or atopic disorders). Our hospital ethic committee approved the design of the study and BAL was obtained after informed consent.

### Monoclonal antibodies (mAb) and flow cytometry

The following mAb and reagents were used in this study: fluorescein isothiocyanate (FITC)–conjugated or peridinin chlorophyll A protein–conjugated anti-CD3 mAb, FITC-conjugated anti-CD45 mAb, FITC-conjugated or phycoerythrin (PE)–conjugated anti-CD56 mAb, PE-conjugated anti-CD16 mAb, PE-conjugated anti-CD122 mAb, and FITC-conjugated antiperforinmAb (all from Becton Dickinson, San Diego, CA). Cells were stained with combinations of appropriate mAb at 4°C for 20 minutes. Stained cells were analyzed on a FACsCalibur flow cytometer using Cell Quest software (BD Biosciences, Mountain View, CA).

### Bronchoalveolar lavage

BAL was obtained as we previously reported [9]. Briefly, bronchoscopy was performed according to standard guidelines [18]. Thirty minutes prior to the procedure patients received 0.5 mg of atropine and 12.5 mg codeine intramuscularly. Local anaesthesia of the oropharynx was achieved by xylocaine instillation until gag reflexes subsided. Bronchoscopy was performed using a Pentax bronchoscope through which 150 ml of normal prewarmed saline in aliquots of 50 ml were instilled into a subsegment of the right middle lobe. BAL fluid (BALF) was then immediately aspirated by gentle hand suction into plastic tubes and kept at 4°C on ice.

BALF was concentrated 10 fold before analysis whilst a great part of the cell pellets were immediately fixed in RNA stabilisation buffer. The total count of nucleated cells was performed as we have recently reported [9]. Differential cell count was determined by cytological examination of at least 500 cells after centrifugation in a cytospin (Shandon) and May-Grünwald-Giemsa staining. Cell percentages were recorded for every patient. The rest of BALF was centrifuged at 400 g for 10 min and the pellet was processed for lymphocyte subset of (Th17) and regulatory T cells (Treg) determination. All BALF were analyzed at the time of processing and only technically appropriate BALF were retrospectively reviewed.

### Flow cytometric assays of the cytotoxicities of BAL cells and purified NK cells

BAL cells and BAL-isolated NK cells were used as effector cells and were cultured for 4 hours at 37°C in complete media, consisting of RPMI 1640, 2 mM-glutamine, 100 units/ml of penicillin, 100 μg/ml of streptomycin, and supplemented with 10% fetal bovine serum (FBS; Gibco BRL, Grand Island, NY) in a humidified incubator containing 5% CO_2_. K562 cells (CCL-243; American Type Culture Collection, Manassas, VA) were used as target cells. Effector and target cells were mixed in 12 × 75–mm round-bottomed polystyrene tubes (Becton Dickinson) at different effector-to-target (E:T) cell ratios. Control tubes including only target cells were also assayed to quantify spontaneous K562 cell death. Tubes were incubated for 4 hours at 37°C in a humidified incubator containing 5% CO_2_.

To determine LAK activities, 200 IU/ml of recombinant IL-2 (BD PharMingen, San Jose, CA) was added to the tubes. Mixed effector and target cells were stained with FITC-conjugated anti-CD45 mAb at 4°C for 20 minutes, washed once in phosphate buffered saline (PBS), resuspended in 0.5 ml of PBS containing 20 μl of 1 μg/ml propidium iodide (BD PharMingen), and incubated at room temperature for 15 minutes. Percentages of dead K562 cells were determined by flow cytometry. Cytotoxicities were calculated by subtracting the percentages of dead K562 cells in control tubes from the percentages of dead cells in sample tubes.

### RT-PCR

Total cellular RNA was extracted from isolated BAL cells and NK cells using RNAzol B (Tel-Test, Friendswood, TX), according to the manufacturer’s instructions. Aliquots (3 μg) of total cellular RNA were transcribed into complementary DNA (cDNA) at 37°C for 1 hour in a total volume of 20 μl using 2.5 units of Moloney murine leukemia virus reverse transcriptase (Roche, Germany). Reverse-transcribed cDNA samples were then added to a PCR mixture consisting of 10× PCR buffer, 0.2 mMdNTPs, 0.5 units of Taq DNA polymerase (Biocare Lab, Tunisia), and 10 pmoles of primers for each gene. The sequences of the primers we used were as follows: for β-actin, 5^′^-CTCCTTAATGTCACGCACGAT-3^′^ (sense) and 5^′^-GTGGGGCGCCCCAG GCACCA-3^′^ (antisense); for perforin, 5^′^-CTGCCGTGGATGCCTATG-3^′^ (sense) and 5^′^-CGGCTCACACTCACAGG-3^′^ (antisense); for granzyme, 5^′^-TACACACAAGA GCTCCAGAGT-3^′^ (sense) and 5^′^-GGGGAAGCTCCATAAATGTCACCT-3^′^ (antisense); for CD122, 5^′^-GGTCACCTGATAGTGGAGAA-3^′^ (sense) and 5^′^-ACCTGAATCCAATTTCACAG-3^′^ (antisense); for c-Kit, 5^′^-TTCTTACCAGGTGG CAAAGGGCATGGCTTTCC-3^′^ (sense) and 5^′^-GTCATACATTTCAGCAGGTGCGTG TTCAGGGC-3^′^ (antisense); and for Flt-3, 5^′^-GAGGACTTGAATGTGCTTACA-3^′^ (sense) and 5^′^-TCCCACAGTAATATTCCATATGA-3^′^ (antisense).

Amplifications were conducted over 28 cycles of 94°C for 1 minute (denaturation), 55°C for 1 minute (annealing), and 72°C for 1 minute (extension). This was followed by an additional extension step at 72°C for 10 minutes in a PCR cycler (Bio-Rad, CA). PCR products were subjected to electrophoresis and visualized by ethidium bromide staining. Densities were analyzed versus β-actin by densitometry using Alpha Ease FC image analysis software (Alpha Innotech, CA). Results are presented as relative gene expression intensities.

### Quantification of RORC/FOXP3 (Th17/Treg) ratios

The expression of mRNA for FOXP3 (Treg) and RORC (Th17) was quantified using the Applied Biosystems 7500 Fast Real-Time PCR System (Applied Biosystems, Foster City, CA, USA) as we have recently reported [9,12]. Amplification of cDNA was performed with the TaqMan Universal PCR Master Mix (2×), No AmpErase UNG (Applied Biosystems). A reaction volume of 25 μl (1.0 μlcDNA) was amplified for 40 cycles of 10s at 95°C and 1 min at 60°C. All samples were analyzed in duplicate, and transcription expression was calculated as a mean and standard deviation (SD). For quantification of cDNA a five-point serially four-fold diluted standard curve was developed from BAL cell cultures stimulated with phytohaemagglutinin (PHA). The mRNA expression of the T cell transcription factors was standardized to 18S (human rRNA) and all results are expressed as a ratio. A coefficient of variance < 15% was accepted as maximum variation among duplicates. The intra-assay variance for 18S was 4.9%, FOXP3 6.1%, RORC 6.2%. Samples revealing an undetectable expression in both duplicates in three subsequent analyses were assigned an expression quantity of zero. Primers and probes for FOXP3 forward GTGGCCCG GATGTG AGAA, reverse GCTGCTCCAG AGACTGTACCATCT, probe CCTCAA GCACTGCCAGGCGGAC; 18S forward CGGCTACCACATCCA AGGAA, reverse GCTGGAATTACCGCGGCT, probe GAGGGCAAGTCTGGTGCCA GCA. HPLC-purified oligonucleotide primers and probes were bought from MedProbe (Oslo, Norway). All in-house designed mRNA amplicons included at least one exon–exon boundary to assure specificity (marked in bold in the sequences above), and reaction concentration was optimized prior to analysis of samples.

### Statistical analysis

The percentages of NK cells were log-transformed for purposes of analysis. All comparisons of percentages of NK cells and of cytotoxicity were made by analysis of covariance after adjusting for age and sex using the Bonferroni correction for multiple comparisons. Comparisons of CD3–CD122^+^ cell percentages and of CD122 surface expression rates among NK cells were made by analysis of variance with the Bonferroni correction for multiple comparisons. *P* less than 0.05 was considered statistically significant. All statistical analyses were performed using SPSS version 14.0 software (SPSS, Chicago, IL).

## Results

### Cell analyses and Th17/Treg ratio in bronchoalveolar lavage

**Figure 1:**
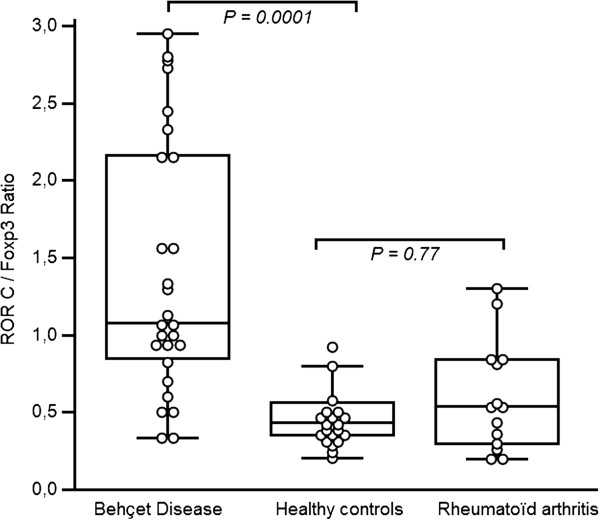
**Transcription factor ratios in BAL cells.** Values were expressed as mean ± SD in the text. *P* is indicated in the Figure. Data are shown as box plots. Each box represents the 25^th^ to 75^th^ percentiles. Lines inside the boxes represent the mean. Whiskers represent the 10^th^ and the 90^th^ percentiles.

BAL fluids recovered from BD patients were more cellular than those from healthy controls and RA patients , containing significantly greater number of lymphocytes (*P*< 0.05) [Table 1]. Expression of retinoid-related orphan receptor C (RORC) (Th17) and forkhead box P3 (FOXP3) (Treg) mRNA transcript were studied in BAL cells from 27 BD patients with pulmonary manifestations, 14 RA patients and 23 healthy controls. FOXP3 was expressed at similar levels between BD patients [1.62 ± 0.60%], RA patients [1.58 ± 0.44%] and healthy controls [1.67 ± 0.59%; *P =* 0.85 and *P =* 0.77]. In contrast, RORC was highly expressed in BAL of BD patients [1.93 ± 0.69%] compared to that of RA patients [0.76 ± 0.22%; *P =* 0.0001] and of healthy controls [0.85 ± 0.38%; *P =* 0.0001]. Th17/Treg (RORC/FOXP3) ratio was increased in BD patients contrasting with values observed in RA patients and in healthy controls (Figure 1).

**Table 1:** Differential cell count

**Diseases**	**Macrophages**	**Lymphocytes**	**Neutrophils**	**Eosinophils**
BD (n = 27)	72.5 ± 7.4	19.7 ± 4.3^†^	7.6 ± 10.9	1.9 ± 4.3
RA (n = 14)	72.6 ± 5,8	14.8 ± 9.7	10.3 ± 11.9	2.4 ± 3.6
HC (n = 23)	78 ± 6.8	12.5 ± 3.4	3.5 ± 1.4	0.5 ± 0.7

BD, Behçet’s disease; HC, Healthy control, RA, rheumatoidarthritis. Values are presented as mean) ± SD. (†): Significance Behçet’s disease versus control subjects and RA patients (*P =* 0.001).

### Reduced numbers of circulating NK cells in BAL cells from BD patients

**Figure 2:**
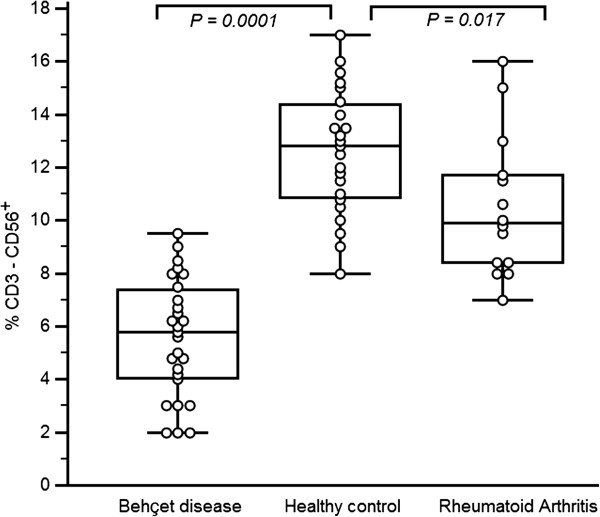
**Reduced numbers of NK cells in BAL of Behçet’s disease.** Freshly isolated bronchoalveolar cells from healthy controls, Rheumatoid arthritis patients and BD patients were stained with fluorescein isothiocyanate–conjugated anti-CD3 and phycoerythrin-conjugated anti-CD56 monoclonal antibodies and then analyzed by flow cytometry. Data are shown as box plots. Each box represents the 25^th^ to 75^th^ percentiles. Lines inside the boxes represent the mean. Whiskers represent the 10^th^ and the 90^th^ percentiles.

The percentages of NK cells in the BAL of BD patients, RA patients and healthy controls were determined by flow cytometry. NK cell percentages were significantly lower in BD patients [5.59% ± 2.22%], than in healthy controls [12.60% ± 2.36%; *P =* 0.0001]. NK cells in RA patients [10.50% ± 2.69%] were expressed at higher level than in BD patients [*P =* 0.0001]. Low and significant difference was found between RA and healthy controls [*P =* 0.017] (Figure 2).

### Impaired cytotoxicity of BAL NK cells in BD patients

**Figure 3:**
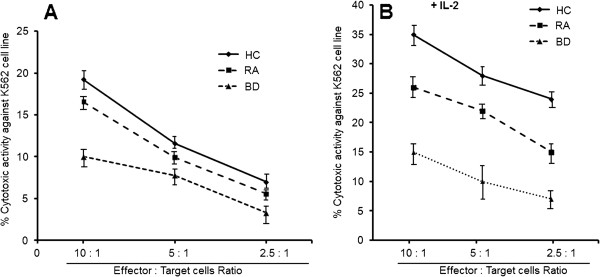
**Decreased cytotoxicity of bronchoalveolar lavage cells from Behçet’s disease patients.** Freshly isolated BAL cells were stained with fluorescein isothiocyanate–conjugated anti-CD45 monoclonal antibody and then cocultured with K562 cells kine for 4 hours. Cytotoxicity was determined as described in patients and methods and is expressed as the percentage of apoptotic K562 cells. For measurement of lymphokine-activated killer (LAK) activity, interleukin-2 (IL-2; 200 IU/ml) was added to cocultures. Cytotoxicity [**A**] and LAK activity [**B**] were determined in BAL cells from 23 healthy controls (HC), 14 patients with RA and 27 patients with Behçet’s disease (BD). Data are Mean ± SD. Cytotoxicity was determined at an effector-to-target (E:T) cell ratio of 10:1, 5:1 and 2.5**:** 1.

To examine the cytotoxic effects of NK cells on K562 cells, BAL from 27 BD patients, 14 RA patients, and 23 healthy controls were used. The cytotoxicities of BAL cells were evaluated by flow cytometry and determined at an effector/target (E:T) cell ratio of 10:1, 5:1 and 2.5:1. The cytotoxicities were significantly lower in BD compared to NK activity in RA patients and in healthy controls (Figure 3A).

Several groups of investigators suggested that NK cytotoxicity was enhanced by IL-2 [19]. LAK activity induced by IL-2 was significantly lower in BD and RA patients than in healthy controls (Figure 3B). Cytotoxicities of BAL cells were significantly correlated with NK cell percentages [NK activity: r = 0.590; *P =* 0.0012]. These findings suggest that impaired cytotoxicity of BAL cells is caused by an NK cell deficiency. Cytotoxicities in BAL from healthy controls were greater at higher E:T cell ratios. This was also the case in BAL from BD and RA patients.

### Intrinsic defects of NK cells in BD patients

**Figure 4:**
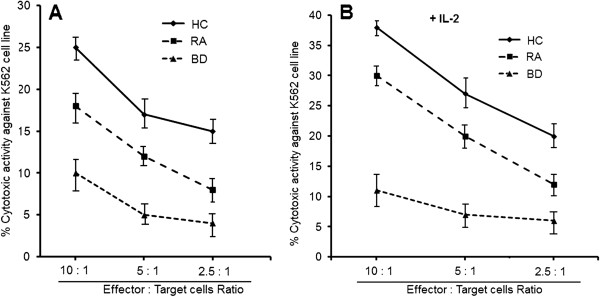
**Decreased cytotoxicity of purified natural killer cells from Behçet’s disease patients.** NK cells were isolated from BAL cells by magnetic-activated cell sorting. Cytotoxicity and lymphokine-activated killer activity were determined as described in Figure 3. Cytotoxicity [**A**] and LAK activity [**B**] of purified NK cells were determined in 5 healthy controls, 3 patients with RA and 7 patients with Behçet’s Disease. Data are shown as box plots. Each box represents the 25^th^ to 75^th^ percentiles. Lines inside the boxes represent the mean. Whiskers represent the 10^th^ and the 90^th^ percentiles. Cytotoxicity was determined at an effector-to-target (E:T) cell ratio of 10:1, 5:1 and 2.5:1.

To examine whether defective killing by NK cells contributes to the impaired cytotoxicity observed in BD patients, we compared the cytotoxicities of purified NK cells obtained from the BAL of 10 BD patients, 7 RA patients and 10 BAL from healthy controls. The cytotoxicities of purified NK cells were determined at an E:T cell ratio of 10/1, 5:1 and 2.5:1. We found that cytotoxicities and LAK activities were significantly lower in BD patients than in healthy controls. These values were significantly lower in RA patients than in healthy controls (Figures 4A and B). Cytotoxicities and LAK activities increased in healthy controls and RA patients when E:T cell ratios increased, which contrasted in BD patients. These results suggest that killing deficits of NK cells contribute to impaired cytotoxicity and LAK activity in BAL from BD patients.

**Figure 5:**
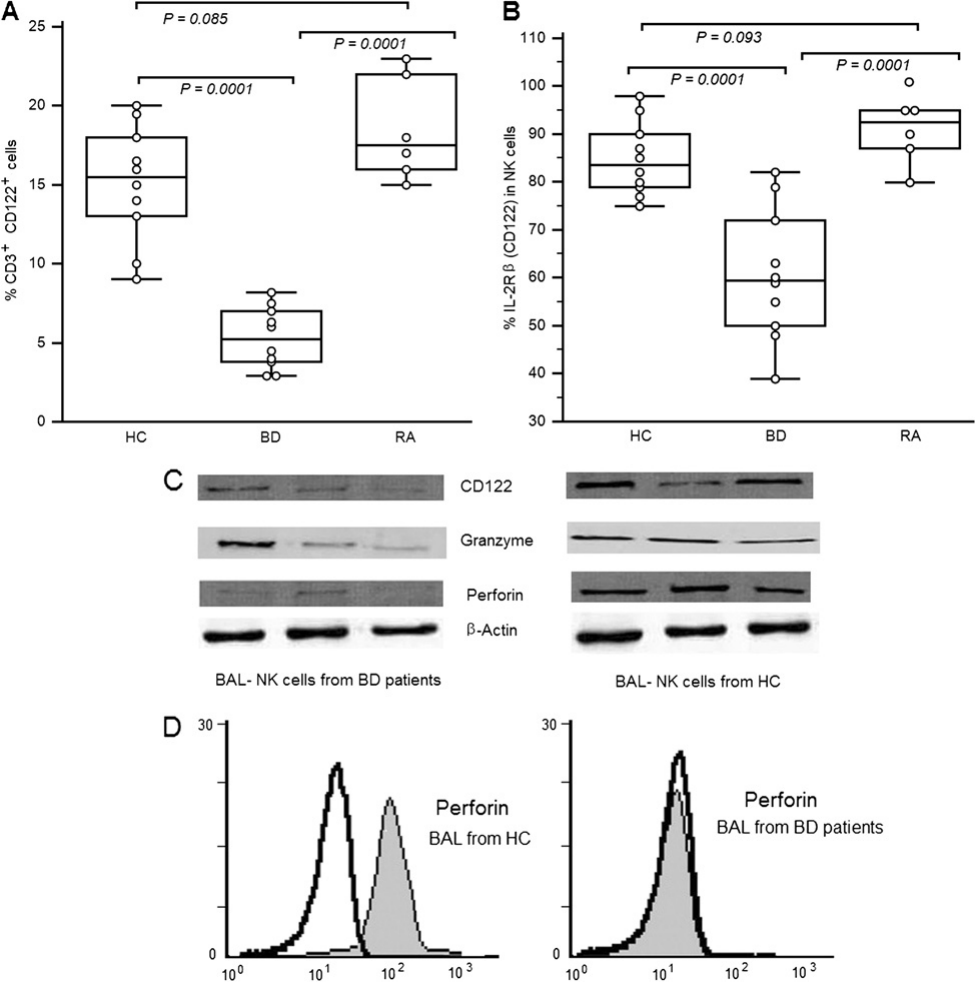
**Reduced expression of CD122 (interleukin-2 receptor β [IL-2Rβ]), perforin, and granzyme in bronchoalveolar lavage from Behçet’s disease patients.** [**A**]: Percentages of CD3^+^CD122^+^ cells in BAL. [**B**] Percentages of IL-2Rβ–expressing cells in natural killer cells from 10 healthy controls 10 patients with BD, and 7 patients with rheumatoid arthritis . Freshly isolated BAL cells were stained with peridinin chlorophyll A protein–conjugated anti-CD3, fluorescein isothiocyanate–conjugated anti-CD56, and phycoerythrin-conjugated anti-CD122 monoclonal antibodies and then analyzed by flow cytometry. Data are shown as box plots. Each box represents the 25^th^ to 75^th^ percentiles. Lines inside the boxes represent the mean. Whiskers represent the 10^th^ and the 90^th^ percentiles. [**C**]: Expression of mRNA for CD122 (IL-2Rβ), perforin, and granzyme in BAL cells and purified NK cells from a healthy control subject and a patient with BD. Total RNA was extracted, reverse-transcribed, and amplified by polymerase chain reaction using primers specific for CD122, perforin, granzyme, and β-actin. PCR products were separated by electrophoresis on 1.0% agarose gels. [**D**]: Perforin expression in NK cells from a healthy control subject and a patient with BD, as determined by intracellular flow cytometry. Shaded regions represent anti-perforin monoclonal antibody (mAb); open regions represent isotype-matched control monoclonal antibody (mAb). Histograms show the CD3^+^CD56^+^ cell population. Results are representative of 3 independent experiments.

The expression of IL-2 receptor β-chain (IL-2Rβ, CD122) is important for the differentiation of NK cells. CD122 is regarded as a marker for NK precursors. CD3^+^CD122^+^ cells include immature and mature NK cells as reported by Huntington et al. [20]. We investigated the proportions of CD3^+^CD122^+^ cells in BAL cells and IL-2Rβ-expressing cells in NK cells. The percentages of CD3^+^CD122^+^ cells and of CD122 expression on NK cells were significantly lower in BD patients [CD3^+^CD122^+^: 5.32 ± 1.92%, *P =* 0.0001; CD122-NK cells: 60.70 ± 13.70, *P =* 0.0001] than in RA patients [CD3^+^CD122^+^: 18.50 ± 3.27%; CD122-NK cells: 92.83 ± 10.11%] and in healthy controls [CD3^+^CD122^+^: 15.10 ± 3.70%; CD122-NK cells: 84.80 ± 7.68%;]. No differences were observed between RA patients and healthy controls [CD3^+^CD122^+^: *P* = 0.085; *P =* 0.093] (Figures 5A and B). We also examined the expression levels of CD122 and of the toxicity-related molecules perforin and granzyme in BAL cells and purified NK cells. Gene transcripts of CD122, perforin, and granzyme were found to be markedly lower in BAL cells and NK cells from BD patients (Figure 5C). Intracellular perforin expression in NK cells was also lower in BD patients than in RA patients and in healthy controls (Figure 5D).

## Discussion

The results of this study demonstrated that BAL cells from BD patients display an inflammatory profile associated with an impairment of NK activity.

The number of Treg cells (FOXP3) was unaltered in BD patients compared to controls, contrasting with an increase in Th17 (RORC) subpopulation. These results suggest that an imbalance of Th17/Treg in BAL cells may be implicated in the pathogenesis of lung involvement in BD. Increased expression of IL-17 in inflammatory sites supports a Th17-mediated inflammatory pathway in several diseases [21-24].

In the present study, we found that NK cell number and cytotoxicity were decreased in BAL from BD patients with pulmonary involvement compared to RA patients and healthy controls. NK cells represent an important population of effector cytotoxic lymphocytes and a major source of pro-inflammatory cytokines. CD3^+^CD122^+^ cell, which include immature and mature NK cells [20], were reduced in BAL from BD patients. Cytotoxicity of BAL purified NK cells were markedly suppressed in BD patients. This activity was not diminished in RA patients. Reduction of cytotoxicity in BD patients resulted from a lowered NK cell number associated with an intrinsic NK cell defect of granzyme expression. This is demonstrated by down-regulation of IL-2Rβ, perforin, and granzyme in NK cells in our patients.

NK cell numbers and activities were persistently low. These data contrast with reported results on peripheral circulation in BD patients, showing increased NK population and high NK activity [11]. This could raise the question of whether local reductions in NK cell number and activity are a consequence of a defective differentiation of hematopoietic stem cells into NK cells, as recently described by Park et al. in systemic lupus patients [11]. This point has to be investigated in a future report. Intensity and quality of the NK cell cytotoxic response depend on the cytokine microenvironment as well as on immune system interactions [25,26]. These interactions include crosstalk between NK cells and dendritic cells (DCs) or T lymphocytes, occurring initially in secondary compartments such as lymph nodes [27]. The phenotyping and functional results from the present study are partially consistent with these findings.

The reduced cytotoxicity in BAL from BD was merely due to low perforin expression. Resting CD56^dim^ NK cells, which account for the majority of circulating NK cells, express intermediate-affinity IL-2Rβ, mediates the induction of LAK activity [28]. In mature NK cells, IL-2Rβ is shared by IL-2 and IL-15, and IL-15 can efficiently induce NK cell proliferation, differentiation, and activation, which markedly increases NK cytotoxicity. However, adjunction of IL-2 to BD NK cells yielded a reduced increase of cytotoxicity compared to controls. This lowered effect of IL-2 on LAK activity in BD is associated with a decrease in IL-2Rβ expression. As IL-2Rβ is critical for LAK activity and NK cell differentiation, down regulation of IL-2Rβ in NK cells from BD patients is consistent with the reduced response of NK cells to IL-2.

A possible explanation is that NK cell depletion in the BD lung patients occurs secondary to disease progression and to the localized important inflammation existing in the lung. However, our present findings suggest that numerical deficiencies and functional defects of NK cells might play an active role in the pathogenesis of BD, rather than being a consequence of the disease process. Indeed, it has been reported that NK cells may control disease flare/remission in BD patients via NK type 2-mediated modulation of the Th1 response [29].

A possible interaction exists between the increased Th17 and the paucity of NK cells. NK cells are able to down-regulate Th17 cell responses to avoid pathologic autoimmunity. In T-bet^-^/^-^mice, the paucity of NK and Natural killer T (NKT) cells contributes to a highly polarized Th17 phenotype [30]. Wu et al. [31] demonstrated that addition of NK cells could inhibit T-bet-deficient, autoreactive Th17 cells in the peripheral immune system. These findings have important implications for the development of Th17 cell-mediated autoimmune pathology and suggest a potential role of NK cells in modulation of Th17 cell-mediated autoimmunity. In the lungs of BD patients the paucity of NK cells is associated with a high Th17 expression. The question remains about IL-17 effect on NK cells.

## Conclusions

Pulmonary manifestations are seen in approximately 25–30% of BD patients and represent the first cause of mortality. Our phenotypic and functional findings suggested that BAL-NK cells from BD patients could have an impaired differentiation. Despite the increasing NK cells in peripheral circulation, which contrasted with failing NK cell activity in the lung, a body of research on inflammatory mechanisms in pulmonary manifestations has to be done. It is still uncertain whether the NK cell in the lung is friend or foe.

## Competing interest

The authors have no conflicts of interest to disclose.

## Authors’ contributions

AH, AB, WK and KH, participated in the design and coordination of the study and to manuscript writing, performed the experiments and analyzed the data. JA participated in the design, coordination of the study and analyzed the data. All authors read and approved the final manuscript.
